# Brentuximab vedotin exerts profound antiproliferative and pro‐apoptotic efficacy in CD30‐positive as well as cocultured CD30‐negative germ cell tumour cell lines

**DOI:** 10.1111/jcmm.13344

**Published:** 2017-09-22

**Authors:** Stefan Schönberger, Cornelius van Beekum, Barbara Götz, Daniel Nettersheim, Hubert Schorle, Dominik T. Schneider, Anna Casati, Rogerio B. Craveiro, Gabriele Calaminus, Dagmar Dilloo

**Affiliations:** ^1^ Department of Paediatric Haematology and Oncology University Children's Hospital Bonn University of Bonn Medical School Bonn Germany; ^2^ Department of Developmental Pathology Institute of Pathology University of Bonn Medical School Bonn Germany; ^3^ Clinic of Paediatrics Municipal Hospital Dortmund Dortmund Germany

**Keywords:** brentuximab vedotin, germ cell tumours, embryonal carcinoma, CD30, monomethyl auristatin E, bystander effect, antibody‐drug conjugate, coculture model

## Abstract

Prognosis in patients suffering from high‐risk, refractory and relapsed germ cell tumours (GCT) often comprising of CD30‐positive embryonal carcinoma (EC) components remains poor. Thus, novel treatment strategies are warranted. The antibody‐drug conjugate (ADC) brentuximab vedotin delivers the potent antimitotic drug monomethyl auristatin E (MMAE) to CD30‐expressing tumour cells. After CD30 binding, internalization and intracellular linker cleavage cytotoxic MMAE can efflux and eradicate neighbouring CD30‐negative cells. To analyse cytotoxicity and a potential bystander effect of brentuximab vedotin in GCT, we established an *in vitro* coculture model mimicking GCT of heterogeneous CD30 positivity and measured cell viability, proliferation and apoptosis after exposure to brentuximab vedotin and unbound MMAE by MTS‐ and flow cytometry‐based CFSE/Hoechst assay. CD30 expression being assessed by quantitative RT‐PCR and immunohistochemistry was apparent in all EC cell lines with different intensity. Brentuximab vedotin abrogates cell viability of CD30‐positive GCT27 EC line exerting marked time‐dependent antiproliferative and pro‐apoptotic activity. CD30‐negative JAR cultured alone barely responds to brentuximab vedotin, while in coculture with GCT27 brentuximab vedotin induces clear dose‐dependent cytotoxicity. Cellular proliferation and cell death are significantly enhanced in CD30‐negative JAR cocultured with CD30‐positive GCT27 compared to JAR cultured alone in proof of substantial bystander activity of brentuximab vedotin in CD30‐negative GCT. We present first evidence that in an *in vitro* model mimicking GCT of heterogeneous histology, brentuximab vedotin exerts potent antiproliferative and pro‐apoptotic activity against both CD30‐positive as well as CD30‐negative GCT subsets. Our results strongly support translational efforts to evaluate clinical efficacy of brentuximab vedotin in high‐risk GCT of heterogeneous CD30 positivity.

## Introduction

GCT thought to arise from primordial germ cells often display a mixed histological phenotype comprising teratoma, seminoma, choriocarcinoma, yolk sac tumour or EC components 1. While the former histologies are CD30 negative, CD30 expression is pathognomonic for EC 2, 3.

Prognosis in patients with seminomatous GCT is excellent and still favourable in non‐seminomatous GCT. In patients with metastatic, refractory or recurrent disease, outcome remains poor 4, 5, 6. These patients often present with GCT of mixed histology comprising EC subpopulations and their survival is below 50% in spite of intensive salvage therapy including high‐dose treatment 4. Thus, novel treatment options are urgently needed.

Today, targeted agents are the focus of anticancer drug development. Cytotoxic agents linked to monoclonal antibodies selectively deliver drugs to target sites enhancing local drug activity while reducing systemic toxicity. Brentuximab vedotin (BV) represents one of these antibody‐drug conjugates (ADC). Brentuximab vedotin targets CD30 with promising clinical responses and good tolerability in patients with refractory Hodgkin (HL) or anaplastic large cell lymphoma 7. It consists of the potent antimitotic drug MMAE coupled by dipeptide linker to the chimeric anti‐CD30 monoclonal antibody cAC10 8, 9. In its antibody‐conjugated form, the toxin is inactive. It exhibits antineoplastic activity after binding to CD30 antigen expressed on the tumour cell surface, internalization through receptor‐mediated endocytosis, linker cleavage and intracellular MMAE release. The toxin MMAE inhibits tubulin polymerization resulting in cell cycle arrest and apoptotic cell death 8, 9. Of note, intracellularly released MMAE effluxes into the extracellular space and may also eradicate neighbouring CD30‐negative cells 8, 9. Based on this bystander effect, brentuximab vedotin represents a highly promising drug for targeted cancer therapy, even in histologically heterogeneous tumours displaying variable CD30 expression.

While first pilot studies evaluating brentuximab vedotin in adults with refractory testicular cancer have been initiated 10, to date, there are no data delineating bystander efficacy of the CD30‐conjugated tubulin toxin on CD30‐negative GCT cells. Here, we present first evidence that in an *in vitro* model mimicking GCT of mixed histology, brentuximab vedotin exerts potent antiproliferative and pro‐apoptotic activity against both CD30‐positive as well as CD30‐negative GCT subsets. Our results provide insights that substantiate early clinical efforts to translate this promising drug into the clinical setting.

## Material and methods

### Cell culture

2102EP, NT2/D1 and NCCIT cells were kindly provided by L. Looijenga (Daniel den Hoed Cancer Center/NL), TCam‐2 by J.Shipley (Institute of Cancer Research, UK), 833KE and GCT27 by T. Müller (Martin‐Luther‐University of Halle, Germany) and B. Köberle (KIT, Germany), respectively. JAR (HTB‐144) and JEG‐3 (HTB‐36) were purchased from American Type Culture Collection. All cell lines are known to be cisplatin sensitive. Cell lines were cultivated as described previously 9, 11, 12.

### Immunohistochemistry

A total of 4 × 10^4^ tumour cells in PBS/1.5% BSA were cytospun at 12000 *g* for 5′ onto glass slides and air‐dried for 15′. Signal detection was performed semiautomatically in the Autostainer 480 S (Medac, Wedel, Germany) using the Bright Vision+ polymer detection system (Medac) and the following settings: anti‐CD30 primary antibody (BER‐H2, dilution 1:200, Dako, Eching, Germany) for 20′, enhancer for 10′, polymer (Poly‐HRP‐Goat anti‐mouse/‐rabbit‐IgG) for 20′, 3,3′‐diaminobenzidine (DAB) (415192F, Medac) for 8′. Nuclei were stained by haematoxylin for 3′.

### Quantitative real‐time RT‐PCR

Quantitative real‐time RT‐PCT (qRT‐PCR) was performed as described previously 13, 14. PCR was performed at 94°C/30′′, 60°C/60′′ for 40 cycles using the ViiA 7 Real‐Time PCR System (Applied Biosystems, Foster City, CA, USA). A melting point analysis was performed to confirm primer specificity.

### Cell viability

Cell viability was assessed by MTS analysis in the ‘CellTiter 96 Aqueous One Solution Cell proliferation Assay’ (Promega, Madison, WI, USA) according to the supplier's instructions. To ensure exponential cell growth over time, 5 × 10^3^ GCT27 and 1 × 10^4^ L540 cells were seeded for 48 hrs, 2.5 × 10^3^ GCT27 and 8 × 10^3 ^L540 cells for 72 hrs and 2 × 10^3^ GCT27 and 5 × 10^3 ^L540 cells for 96 hrs in a 96‐well plate in 100 μl medium at 37°C. After 4 hrs, 250 ng/ml BV and 100 pM MMAE (both kindly provided by Seattle Genetics, Bothell, WA, USA) or the vehicle PBS was added.

### Assessment of viable cell numbers, proliferation and apoptosis by flow cytometry

1 × 10^5^ GCT27, 1 × 10^5^ NCCIT and 0.5 × 10^4^ JAR cells were labelled with CFSE (Invitrogen, Waltham, MA, USA) according to supplier's instructions and cultivated either in coculture or separately. After 12 hrs, 100 pM MMAE or 250, 500 or 1000 ng/ml BV or PBS as control was added. For flow cytometric enumeration of viable cell number and proliferation analysis, cells were washed after 24–96 hrs and resuspended in 200 μl PBS/2%FBS containing 1 μg/ml Hoechst 33258 (Sigma‐Aldrich, Munich, Germany) for assessment of dead cells. Cellular proliferation was traced by progressive carboxyfluorescein succinimidyl ester (CFSE) dilution.

### Statistical analysis

Calculations of mean values, standard deviation and *P*‐values were performed using Microsoft Excel and GraphPad Prism. For determination of statistical significance between groups, the two‐tailed Student′s *t*‐test was used. *P*‐values < 0.05 were considered statistically significant.

## Results

### EC cell lines express CD30 mRNA and protein

**Figure 1:**
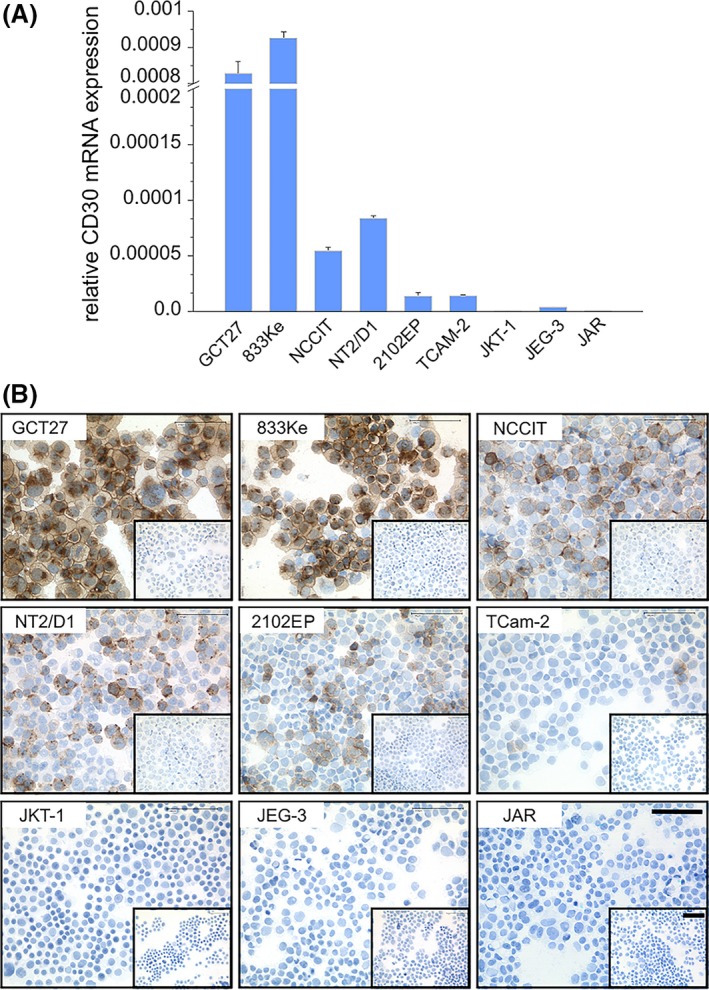
Embryonal carcinoma (EC) cell lines express CD30 mRNA and protein. (**A**) Quantitative Real‐Time PCR analysis of *CD30* mRNA expression in nine GCT cell lines. Relative *CD30* expression levels were normalized against GAPDH and presented as 2^−Δct^ values. Samples were analysed in triplicates. (**B**) Immunohistochemistry analysis of CD30 expression in the same nine GCT lines by DAB and haematoxylin stain. Original magnification: 400x. Scale bar = 200 μm. For each cell line, the control stain for the secondary antibody is illustrated in the frame in bottom right corner.

The EC cell lines GCT27 and 833Ke exhibit the highest *CD30* mRNA levels. In NCCIT, NT2/D1 and 2102EP mRNA levels are 1–2 two log lower (Fig. 1A). *CD30* mRNA expression in the seminoma line TCam‐2 resembles 2102EP, while it is low in choriocarcinoma‐derived JEG‐3 and negligible in JKT‐1 (non‐seminoma) and JAR (choriocarcinoma).

Likewise in immunohistochemical analysis (Fig. 1B), the EC cell lines GCT27 and 833Ke present strong homogenous membranous staining of CD30 while NCCIT, NT2/D1 and 2102EP exhibit focal CD30 positivity. Sparse CD30 staining is demonstrated in the seminoma line TCam‐2, while JKT‐1 or the choriocarcinoma lines JEG‐3 and JAR are CD30‐negative.

### Time‐dependent reduction of cell viability in CD30‐positive GCT27 and CD30‐positive L540

**Figure 2:**
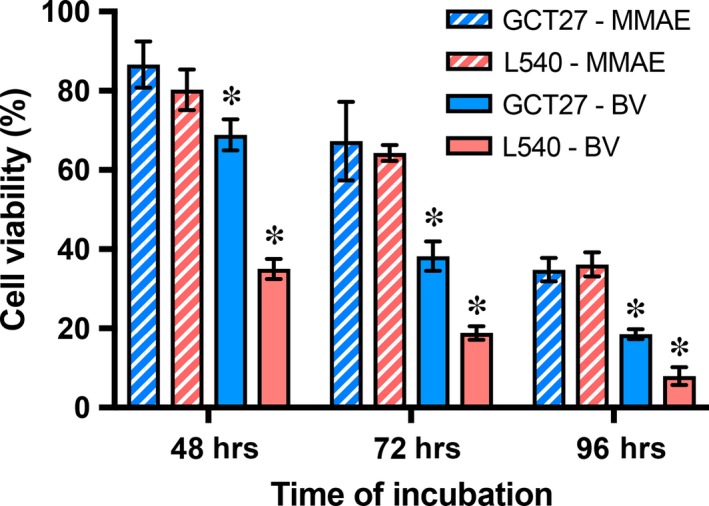
Time‐dependent reduction of cell viability in CD30‐positive GCT27 and CD30‐positive L540 after exposure to tubulin toxin monomethyl auristatin E (MMAE) and brentuximab vedotin (BV). The embryonal carcinoma cell line GCT27 and the Hodgkin lymphoma cell line L540 were incubated with 250 ng/ml brentuximab vedotin or unbound MMAE over time. Cell viability was assessed by MTS assay and expressed relative to untreated control. Statistical differences between the MMAE‐ and BV‐exposed cell line are marked by asterisk (*P* < 0.01). Data are presented as mean ± S.D. of 2 independent experiments performed in sextuplets.

Cell viability was assessed in the CD30‐positive EC line GCT27 in comparison with CD30‐positive Hodgkin line L540 by MTS assay (Fig. 2). After 48 hrs, 100 pM of the unbound cytotoxin MMAE reduces cell viability to 86.6 ± 5.8% in GCT27 and to 80.3 ± 5.1% in L540 and drops to 34.8 ± 3.0% in GCT27 and to 36.2 ± 3.1% in L540 after 96 hrs. Also the antineoplastic efficacy of brentuximab vedotin is time dependent. Viability of CD30‐positive GCT27 cells declines from 68.9 ± 3.9% at 48 hrs to 18.5 ± 1.3% at 96 hrs. At that time, residual cell viability in L540 cells is 7.9 ± 2.2%.

### Brentuximab vedotin reduces viable cell number in CD30‐positive but not CD30‐negative GCT

**Figure 3:**
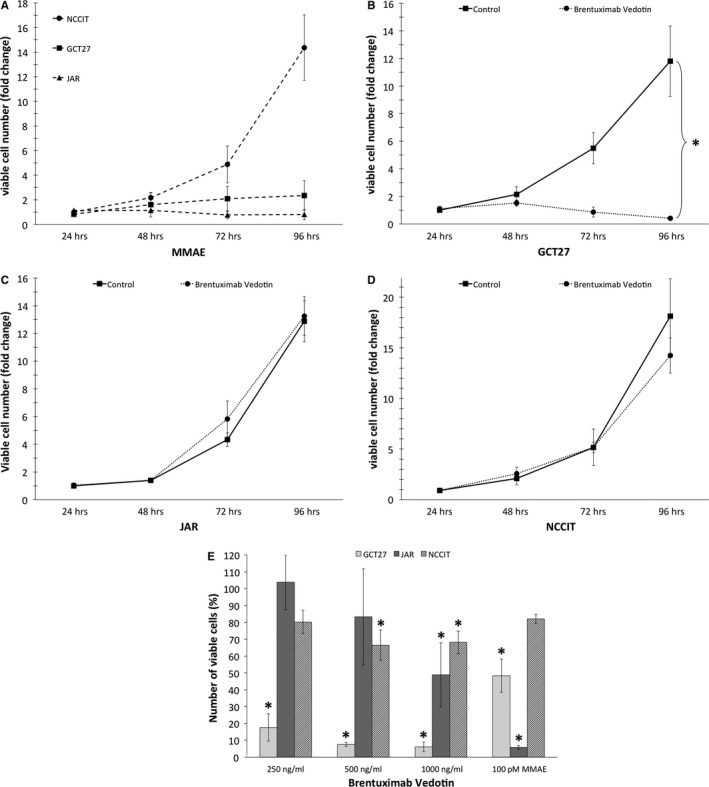
Brentuximab vedotin reduces viable cell number in CD30‐positive but not CD30‐negative GCT cells. The CD30‐positive EC cell lines GCT27 and NCCIT as well as the CD30‐negative choriocarcinoma line JAR were treated with MMAE (**A**) or brentuximab vedotin (**B**–**D**). GCT27 (**B**), JAR (**C**) and NCCIT (**D**) were exposed to 250 ng/ml brentuximab vedotin. After 24, 48, 72 and 96 hrs of culture, cells were resuspended in equal volume for analysis. Viable Hoechst‐negative cells were enumerated for 180 sec. by flow cytometry and are represented as multiples (x‐fold) of the untreated control obtained at 24 hrs. To further evaluate dose‐dependent effects to brentuximab vedotin, the three cell lines were exposed for 96 hrs to 250, 500 and 1000 ng/ml of the ADC as well as 100 pM MMAE (**E**). Enumerated viable Hoechst‐negative cells are expressed in percent of the untreated control at 96 hrs. Statistical differences between untreated control and drug‐exposed cell lines are marked by asterisk (*P* < 0.05). Data represent the mean ± S.D. of two independent experiments performed in duplicates (NCCIT, JAR) or triplicates (GCT27).

Viable cells were enumerated by flow cytometry with the cell count after 24 hrs serving as reference point for subsequent cellular gain to analyse time dependency. GCT cell lines are of variable MMAE sensitivity (Fig. 3A). During 96 hrs in the presence of 100 pM MMAE, viable cell numbers increase only twofold (2.34 ± 1.20) in CD30‐positive GCT27 cells. In the CD30‐negative choriocarcinoma line JAR, cellular growth is entirely arrested by exposure to MMAE with 0.80 ± 0.42‐fold viable cells of the initial 24 hrs count (Fig. 3A). In contrast, the EC line NCCIT of heterogeneous CD30 positivity exhibits very low MMAE sensitivity. Thus viable cell numbers increase 14.4‐fold (14.37 ± 2.66) over 96 hrs in spite of treatment with 100 pM MMAE.

The proliferative capacity is comparable in the three GCT cell lines. Responsiveness to brentuximab vedotin depends on CD30 expression as well as MMAE sensitivity. During 96 hrs, untreated CD30‐positive GCT27 cells (Fig. 3B) expand 11.8‐fold (11.80 ± 2.55) while exposure to 250 ng/ml brentuximab vedotin reduces the viable cell count to less than half (0.41 ± 0.11) of the initial 24‐hr count. The CD30‐negative choriocarcinoma line JAR (Fig. 3C) shows a similar proliferative potential to GCT27 but is unaffected by 250 ng/ml brentuximab vedotin. In 96 hrs, viable cells increase 13‐fold in the absence (12.81 ± 1.61) and in the presence (13.17 ± 1.39) of brentuximab vedotin. Untreated NCCIT presents the highest proliferative activity with a 18.2‐fold (18.24 ± 3.70) rise in viable cell count after 96 hrs (Fig. 3D). Targeting CD30 expressed on the EC line NCCIT with 250 ng/ml brentuximab vedotin attenuates proliferation slightly with a 14.3‐fold (14.34 ± 1.74) rise in viable cell numbers (*P* = 0.1).

To evaluate dose‐dependent effects to brentuximab vedotin, GCT27, JAR and NCCIT cell lines were exposed for 96 hrs to 250 ng/ml, 500 ng/ml and 1000 ng/ml ADC (Fig. 3E). As already shown by the proliferation kinetics above, MMAE sensitivity differs greatly between the GCT lines. The CD30‐negative choriocarcinoma cell line JAR exhibits the highest sensitivity, with only 5.8 ± 1.2% cells surviving compared to the untreated control after exposure to 100 pM MMAE. The EC cell lines are less MMAE sensitive with suppression of viable cell number to 48.4 ± 9.8% in GCT27 yet only 82.1 ± 2.7% in NCCIT demonstrating that this highly proliferative EC line exhibits low susceptibility to the spindle toxin. In the MMAE‐sensitive and highly CD30‐positive cell line GCT27, dose escalation of brentuximab vedotin results in profound suppression of viable cell numbers ranging from 17.6 ± 8.2% of the untreated control at 250 ng/ml to 6.1 ± 2.9% at 1000 ng/ml. Of note, even in NCCIT exhibiting low responsiveness to MMAE and only heterogeneous CD30 expression, brentuximab vedotin attenuates viable cell count to 66.5 ± 9.0% of the untreated control at 500 ng/ml (*P* = 0.012) and 68.1 ± 6.7% at 1000 ng/ml (*P* < 0.001).

Using linear regression analysis, we calculated the IC_50_ values of brentuximab vedotin in the three different cell lines: CD30‐positive and MMAE‐sensitive GCT27 cells exhibit strong vulnerability to the ADC with an IC_50_ value of 219.5 ng/ml. The EC cell line NCCIT presenting low *CD30* mRNA expression and only focal CD30 positivity by immunohistochemistry (Fig. 1) as well as low MMAE sensitivity (Fig. 3A) are less susceptible but still sensitive for brentuximab vedotin. Here, the extrapolated IC_50_ value of 1400.8 ng/ml indicates the need for higher concentrations of the ADC to induce a cytotoxic effect. In the CD30‐negative but highly MMAE‐sensitive choriocarcinoma cell line JAR, IC_50_ was 1013.0 ng/ml.

### Brentuximab vedotin exerts pronounced bystander activity on MMAE‐sensitive, CD30‐negative GCT cells in coculture with CD30‐positive embryonal carcinoma

**Figure 4:**
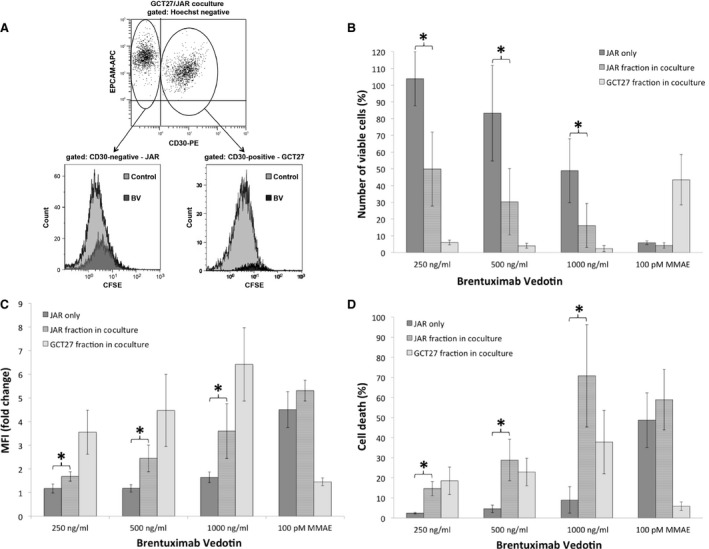
Brentuximab vedotin exerts pronounced bystander activity on MMAE‐sensitive, CD30‐negative GCT cells in coculture with CD30‐positive embryonal carcinoma. For determination of bystander efficacy after drug exposure, cells were stained with CSFE and anti‐CD30.PE (BER‐H2, eBiosience/Germany). CSFE‐labelled CD30‐negative JAR cells were incubated alone or cocultured with the CD30‐positive EC line GCT27. After 96 hrs of drug exposure, cell cultures were resuspended in equal volume and acquired for 180 sec. by flow cytometry. CD30‐negative JAR cells are EPCAM positive (*y*‐axis) but CD30 negative (*x*‐axis) while GCT27 cells are EPCAM and CD30 positive. Hoechst‐negative viable CD30‐positive and CD30‐negative subpopulations were assessed separately after gating on the respective cell fraction (**A**). Viable cell numbers are expressed in per cent of untreated control (**B**). Proliferation is investigated by CSFE dilution upon cellular division. To indicate inhibition of proliferation MFI of experimental conditions was normalized to the MFI of untreated cells and presented as x‐fold MFI (**C**). Cell death was quantified as the proportion of Hoechst‐positive cells of the entirety of acquired cells (**D**). Each column represents the mean ± S.D. of two to three independent experiments performed in duplicates. Statistical differences between results of JAR single‐ and coculture are indicated by asterisk (*P* < 0.05).

Due to its low MMAE sensitivity, NCCIT, although comprising both CD30‐positive and CD30‐negative GCT cells, is not suitable for delineation of a potential bystander activity of brentuximab vedotin. Instead, we established a coculture model of two intensely MMAE‐sensitive GCT cell lines differing in their CD30 expression. For this purpose, CSFE‐labelled CD30‐negative and highly MMAE‐sensitive JAR cells were cocultured with the CD30‐positive and MMAE‐sensitive GCT27 line. After 96 hrs, CD30‐positive and CD30‐negative cell fractions were assessed separately by flow cytometry (Fig. 4A). Viable cell numbers after drug exposure were calculated in relation to the respective untreated control (Fig. 4B).

Viable cell numbers of CD30‐negative JAR cells being cultured alone for 96 hrs and treated with 250 ng/ml brentuximab vedotin are comparable to untreated control. In contrast, in coculture with the CD30‐positive GCT27 cell line, only 49.9 ± 22.1% of the JAR cells survive (*P* = 0.01). Cocultured viable JAR cells decline further to 30.3 ± 19.8% at 500 ng/ml and to 16.1 ± 13.1% at 1000 ng/ml brentuximab vedotin which is significantly lower than in JAR cells cultured alone with 83.3 ± 28.6% at 500 ng/ml (*P* = 0.02) and 48.9 ± 19.1% at 1000 ng/ml Brentuximab Vedotin (*P* = 0.02). Also, brentuximab vedotin reduces cell numbers of the CD30‐positive GCT27 fraction to 6.0 ± 1.4% of the untreated control at 250 ng/ml, to 4.1 ± 1.5% at 500 ng/ml and to 2.3 ± 1.8% at 1000 ng/ml.

To assess the relative contribution of antiproliferative effects to the overall cytoreductive activity of brentuximab vedotin, the mean fluorescence intensity (MFI) of viable CFSE‐stained cells was obtained for CD30‐positive and CD30‐negative tumour cells (Fig. 4C). Inhibition of cellular division results in maintenance of higher CFSE levels expressed as MFI multiples of untreated cells. Highest MFI are observed in CD30‐positive GCT27 cells ranging from 3.6‐ (3.55 ± 0.93) to 6.4‐fold (6.42 ± 1.55) after treatment with 250–1000 ng/ml brentuximab vedotin. In coculture with CD30‐positive GCT27 cells, MFI in CD30‐negative JAR cells are also higher than in untreated control, namely 2.5‐fold (2.45 ± 0.56) and 3.6‐fold (3.60 ± 1.16) after exposure to 500 ng/ml and 1000 ng/ml brentuximab vedotin, respectively. In contrast, when CD30‐negative JAR cells are cultured alone in the presence of 250 ng/ml or 500 ng/ml brentuximab vedotin, MFI are similar to the untreated control and only 1.6‐fold (1.65 ± 0.22) higher at 1000 ng/ml brentuximab vedotin. Thus for each brentuximab vedotin dose level, inhibition of proliferation is significantly more pronounced when CD30‐negative JAR cells are in the presence of GCT27 compared to JAR cells cultured alone (*P* < 0.05).

Cell death (Fig. 4D) was quantified as the proportion of Hoechst‐positive cells of the entirety of acquired cells. In CD30‐negative JAR cells cultured alone, the amount of Hoechst‐positive cells does not significantly differ from the untreated control (data not shown). In contrast, in the fraction of cocultured JAR cells, brentuximab vedotin induces cell death from 14.5 ± 3.5% to 70.8 ± 25.5% after incubation with 250–1000 ng/ml brentuximab vedotin. Under the same conditions, apoptosis of CD30‐positive GCT27 cell fraction ranges from 18.5 ± 6.9% to 37.8 ± 15.7% with high standard deviations as residual cell numbers are already exceedingly low at 96 hrs.

## Discussion

In cancer of mixed histologies such as GCT, the development of antibody‐conjugated drugs faces the challenge of cytotoxin delivery to the entirety of neoplastic subpopulations. In the setting of variable target expression, potent bystander mechanisms constitute the key to therapeutic success.

In testicular cancer, persistence of CD30 expression after first‐line chemotherapy is associated with a particularly poor prognosis 15. Thus, in these EC patients, the CD30‐ADC brentuximab vedotin delivering the spindle‐toxin MMAE to CD30‐positive tumour cells present a promising salvage therapy. Screening a panel of GCT cell lines for our *in vitro* model, we confirm that CD30 is intensely detectable in cell lines derived from EC whereas it is sparse in seminoma and absent in choriocarcinoma. We also demonstrate different sensitivity to the spindle‐toxin MMAE both in its CD30‐conjugated and unbound form. These patterns correspond to the range of responsiveness previously described for lymphoma 8, 9. Most importantly, we delineate pronounced antineoplastic efficacy of brentuximab vedotin in CD30‐positive GCT as well as significant bystander activity in CD30‐negative GCT cocultured with CD30‐positive cells.

Of note, brentuximab vedotin‐mediated cytotoxicity is already substantial in CD30‐positive EC line GCT27 at concentrations as low as 250 ng/ml. Further dose escalation to 1000 ng/ml corresponding to patients` plasma levels under standard brentuximab vedotin administration 16 enhances the antiproliferative and pro‐apoptotic effect. In contrast, the CD30‐negative MMAE‐sensitive choriocarcinoma cell line JAR does not respond up to 1000 ng/ml brentuximab vedotin. Slight antiproliferative and pro‐apoptotic effects at this concentration after 96 hrs occur most likely due to successive hydrolytic release of the tubulin‐toxin MMAE. Of note, expression of different cathepsins known to mediate MMAE cleavage from the ADC is observed in a rodent choriocarcinoma cell line 17.

In coculture of JAR with CD30‐positive EC line mimicking GCT of mixed histology, bystander efficacy is already discernable at brentuximab vedotin concentrations as low as 250 ng/ml, too. We observed marked cytotoxicity on cocultured CD30‐negative JAR cells that exceeded the antineoplastic effect observed in single culture at all dose levels. This cumulative cytoreduction results from significant inhibition of cellular expansion as well as induction of cell death in keeping with the disruption of the microtubule architecture.

After internalization of brentuximab vedotin bound to CD30‐positive tumour cells and endosomal cleavage of MMAE 8, bystander activity is attributed to diffusion of hydrophobic MMAE into the extracellular space. Thus, MMAE exert its antineoplastic activity also on CD30‐negative tumour cells in the vicinity 8, 18. Alternatively, CD30‐positive membranous vesicles liberated from the surface of CD30‐positive tumour cells may translocate the target antigen to CD30‐negative bystander cells and facilitates brentuximab vedotin binding and consecutive cellular kill as recently shown in HL 19. Whether in GCT such tumour‐derived vesicles exist has to be elucidated. Moreover, infiltrating immune cells might also contribute to bystander activity. In this context, it is of interest that in testicular cancer, activated B cells infiltrate the tumour site as part of the inflammatory response 20.

The different experimental models of bystander activity substantiate the clinical observation that in lymphoma patients, brentuximab vedotin has shown clinical efficacy regardless of the level of CD30 expression including CD30‐negative tumour cells 21, 22. Brentuximab vedotin responsiveness is not only determined by density and distribution of CD30 expression but also by the rate of CD30 internalization, speed of lysosomal processing and intracellular MMAE release 9, 23.

To date, nine patients with refractory GCT were treated with brentuximab vedotin as third‐line therapy in a phase II clinical trial 10. The fact that the two patients who achieved CR and VGPR had suffered from GCT of mixed histology substantiates our *in vitro* observation of potent brentuximab vedotin‐mediated bystander activity that has the potential to eradicate even bulky tumours.

In the remaining patients, serum tumour marker levels were attenuated albeit transiently indicating that resistance mechanisms may limit ADC efficacy. Indeed, resistance to brentuximab vedotin particularly in heavily pre‐treated patients is of concern. In GCT, substantial down‐regulation of CD30 surface antigen after chemotherapy has been observed 24, 25. A potential strategy to counteract this mechanism is the administration of Histone deacetylase inhibitors to induce CD30 expression 26. Of note, the histone deacetylase inhibitor romidepsin induces apoptosis and cell cycle arrest of GCT *in vitro* and *in vivo* 27, especially in combination with the small‐molecule inhibitor JQ1 which selectively binds the ‘BET’ family of bromodomain proteins 28. Beyond loss of target expression, responsiveness to brentuximab vedotin may also be due to MMAE resistance which in HL has been attributed to the multidrug resistance protein 1 (MDR1) 29. In testicular GCT, MDR1 expression is associated with advanced stage of disease and platinum resistance 30. However, MDR1 is not expressed in the GCT line NCCIT exhibiting low MMAE responsiveness *in vitro* (data not shown). Still in clinical studies evaluating efficacy of brentuximab vedotin in GCT, prospective assessment of MDR1 expression may be considered.

Multimodality regimens are the hallmark of effective cancer therapy. First experiences indicate that the combination of brentuximab vedotin with cisplatin or radiotherapy ‐ treatment modalities of proven efficacy in GCT ‐ might be well tolerated 31, 32. The combination of established treatment regimens with targeted drug delivery has the potential to enhance antineoplastic efficacy while limiting systemic and long‐term side effects related to chemotherapy.

Here, we document pronounced antiproliferative as well as pro‐apoptotic activity of brentuximab vedotin in GCT. Brentuximab vedotin effectively targets the tubulin‐toxin MMAE to CD30‐expressing GCT with substantial cytotoxicity on CD30‐negative bystander cells. These potent bystander mechanisms are a prerequisite for therapeutic success in tumours of mixed histologies such as GCT.

## Conflict of interest

The authors confirm that there are no conflict of interests.
